# Association of Localized Hypersensitivity and In-Stent Neoatherosclerosis with the Very Late Drug-Eluting Stent Thrombosis

**DOI:** 10.1371/journal.pone.0113870

**Published:** 2014-11-25

**Authors:** Kyohei Yamaji, Shunsuke Kubo, Katsumi Inoue, Kazushige Kadota, Shoichi Kuramitsu, Shinichi Shirai, Kenji Ando, Masakiyo Nobuyoshi, Kazuaki Mitsudo, Takeshi Kimura

**Affiliations:** 1 Division of Cardiology, Kokura Memorial Hospital, Kitakyushu, Japan; 2 Department of Cardiology, Kurashiki Central Hospital, Kurashiki, Japan; 3 Division of Laboratory Medicine, Kokura Memorial Hospital, Kitakyushu, Japan; 4 Department of Cardiovascular Medicine, Kyoto University Graduate School of Medicine, Kyoto, Japan; University of Messina, Italy

## Abstract

**Background:**

Localized hypersensitivity reaction, delayed arterial healing, and neoatherosclerosis inside the stent have been suggested as the underlying pathologic mechanisms of very late stent thrombosis (VLST) of drug-eluting stent (DES). The present study sought to explore the prevalence of inflammatory cell infiltrates and evidence for fragments of atherosclerotic plaques in the aspirated thrombi in patients with DES VLST.

**Methods and Results:**

From April 2004 to September 2012, 48 patients with stent thrombosis (ST) of DES underwent thrombus aspiration with retrieved material sufficient for the histopathologic analysis; early ST (EST, within 30 days): N = 17, late ST (LST, between 31 and 365 days): N = 7, and very late ST (VLST, >1 year): N = 24. Eosinophil fraction in the aspirated thrombi was significantly higher in patients with VLST (8.2±5.7%) as compared with those with EST (4.3±3.0%) and LST (5.5±3.8%) (P = 0.03). Eosinophil fraction in the aspirated thrombi was significantly higher in 12 VLST patients with angiographic peri-stent contrast staining (PSS) and/or incomplete stent apposition (ISA) by intravascular ultrasound than in 12 VLST patients without PSS or ISA (10.6±6.1% versus 5.8±4.1%, P = 0.03). Evidences for fragments of atherosclerotic plaques in the aspirated thrombi were observed only in 3 (13%) out of 24 patients with DES VLST.

**Conclusions:**

Eosinophil fraction in the aspirated thrombi was significantly higher in patients with DES VLST as compared with those with EST and LST. Evidences for fragments of atherosclerotic plaques were relatively uncommon in patients with DES VLST.

## Introduction

Very late stent thrombosis (VLST) was a rare but life-threatening complication, [1]–[3] occurring at the rates of 0.2–0.6%/year without attenuation up to at least 5 years after the implantation of the first-generation drug-eluting stents (DES) as compared with 0.05%/year after bare-metal stent (BMS). [4]–[8] Several studies have suggested possible pathologic mechanisms for this late adverse event. Localized hypersensitivity reaction with extensive vasculitis consisting predominantly of lymphocytes and eosinophils was observed in a patient suffering from VLST. [9] Incomplete stent apposition (ISA) with positive remodeling by intravascular ultrasound (IVUS) was highly prevalent in patients with DES VLST, [10]–[13] and appeared to be associated with higher fraction of eosinophil in the aspirated thrombi. [14] An autopsy case with sirolimus-eluting stent (SES) thrombosis demonstrated abnormal angiographic finding called peri-stent contrast staining (PSS) with a histopathologic evidence of chronic inflammation and hypersensitivity vasculitis. [15] PSS characterized by ISA or multiple cavities between and outside the strut, was associated with subsequent target-lesion revascularization and VLST. [16]–[18] Delayed arterial healing manifested by persistent fibrin deposition and incomplete reendothelialization could be another underlying mechanism of VLST. [19], [20] The majority of stents with delayed arterial healing were those deployed for off-label indications, and underlying mechanisms for VLST in those patients were localized hypersensitivity with SES and malapposition secondary to excessive fibrin deposition with paclitaxel-eluting stents (PES). [21] In a postmortem study, neoatherosclerosis inside the stent occurred significantly earlier in DES lesions as compared with BMS lesions, and was suggested to be related to VLST. [22] Therefore, localized hypersensitivity reaction, delayed arterial healing, and neoatherosclerosis inside the stent have been suggested as underlying pathologic mechanisms of DES VLST.

In an attempt to further explore the mechanisms of VLST, we conducted a retrospective pathologic analyses of aspirated thrombi at the time of DES thrombosis from 2 Japanese centers, evaluating inflammatory cell infiltrates and evidence for fragments of atherosclerotic plaques.

## Methods

### Patient Population

**Figure 1:**
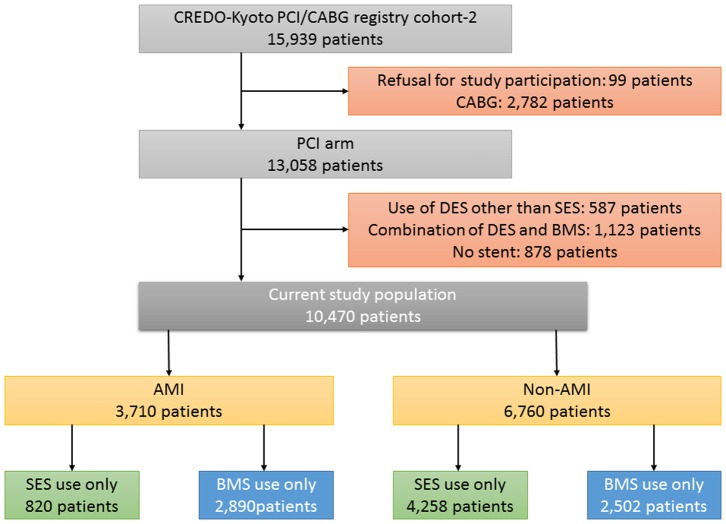
Study flow chart of patients with drug-eluting stent thrombosis who underwent thrombus aspiration with retrieved material sufficient for the histopathologic analysis. DES  =  drug-eluting stents, EST  =  early stent thrombosis, LST  =  late stent thrombosis, and VLST  =  very late stent thrombosis.

From April 2004 to September 2012, we identified 105 patients who underwent percutaneous coronary intervention (PCI) for angiographically confirmed definite stent thrombosis (ST) of DES from the databases at Kokura Memorial Hospital (N = 39) and Kurashiki Central Hospital (N = 66). Thrombus aspiration using manual aspiration catheters was performed at the time of PCI in 75 patients. The amount of aspirated thrombi was sufficient for the histopathologic analysis in 48 patients, who constituted the current study population. Regarding the timing after the index DES implantation, there were 17 patients with early ST (EST, within 30 days), 7 patients with late ST (LST, between 31 and 365 days), and 24 patients with very late ST (VLST, >1 year) (Figure 1). Median durations between the index DES implantation procedure and ST were 5 (interquartile range [IQR]: 3–13) days for EST, 61 (IQR: 45–286) days for LST, and 1349 (IQR: 1032–1886) days for VLST. The types of the thrombosed stents included SES (Cypher, Cordis, Johnson & Johnson) in 29 patients, PES (Taxus, Boston Scientific) in 8 patients, zotarolimus-eluting stents (ZES, Endeavor, Medtronic) in 4 patients, everolimus-eluting stents (EES, Xience, Abbott vascular, or Promus, Boston Scientific) in 3 patients, and biolimus-eluting stents (BES, Nobori, Terumo, or Biomatrix, Biosensors) in 4 patients.

The study protocol was approved by the institutional review board of both Kokura Memorial Hospital and Kurashiki Central Hospital. Because of retrospective enrollment, written informed consents from the patients were waived. All data provided by each center were anonymized before the analysis.

### Definitions

Clinical information was obtained from the medical records in each center. Detailed definitions of clinical variables were described in the previous report. [23] In brief, diabetes mellitus was diagnosed when a patient was treated with insulin or oral hypoglycemic drugs, or when casual levels of plasma glucose were higher than 200 mg/dl, fasting levels of plasma glucose were higher than 126 mg/dl or HbA1c was higher than 6.5% in patients without treatment with insulin or oral hypoglycemic drugs. Chronic renal failure was defined as estimated glomerular filtration rates less than 30 ml/min/1.73 m^2^. Current smoking included current smokers and ever smokers who quit smoking within 1 month. Definite ST was defined as angiographic evidence of thrombus in association with the symptoms and signs of acute coronary syndrome according to the Academic Research Consortium definition. [24]

### Thrombus Aspiration

All patients received 200 mg of aspirin and either 300 mg of clopidogrel or 200 mg of ticlopidine before PCI, unless maintenance doses of dual antiplatelet therapy (DAPT) were administered before the onset of ST. An intra-arterial bolus of heparin (10000 IU) and an intracoronary bolus of nitroglycerin (250 µg) were administered before coronary angiography. Coronary angiography was performed using 6 French or 7 French guiding catheters. Then a 0.014-inch guidewire was passed through the occluded coronary segment, followed by thrombus aspiration using manual aspiration catheters such as Thrombuster (Kaneka Corporation, Japan), Rebirth Pro (Goodman Corporation, Japan), and Eliminate (Terumo Corporation, Japan). The choice of the aspiration devices was left to the discretion of the operators.

### Histopathological Analysis

The aspirated thrombi were fixed in 10% buffered formalin and embedded in paraffin. Paraffin blocks of the thrombi were sectioned using a rotary microtome and stained with luna and hematoxylin and eosin. An experienced pathologist (Inoue K) performed the histopathologic analysis in a blinded manner regarding the timing of ST onset. Using Luna stain, we selected up to five (mean 3.9±0.91; ≧4 in 39 patients; ≤3 in 9 patients) high-power fields (0.145 mm^2^/field) for evaluating inflammatory cell infiltrates. The number of eosinophils and total white blood cells per mm^2^ were counted to calculate eosinophilc fraction in the aspirated thrombus. In consistent with the previous study, evidences for fragments of atherosclerotic plaques were regarded as present when at least one of the following pathologic findings such as a thin fibrous cap, foamy macrophages, or cholesterol crystals was identified using hematoxylin and eosin stain. [23]

### Angiographic Analysis

Angiographic analysis was conducted by the 2 investigators (Yamaji K and Kubo S). All the study patients were confirmed to fulfill the definition of definite ST. According to the institutional protocols, patients in Kokura Memorial Hospital were encouraged to undergo follow-up angiography at 6 and 12 months, while those in Kurashiki Central Hospital at 8 and 24 months, respectively. We evaluated stent fracture and PSS outside the stent at the time of follow-up angiography or at the time of ST after thrombus aspiration and/or balloon angioplasty. Stent fracture was defined as complete or partial separation of stent segments observed by plain fluoroscopy without contrast injection. [25], [26] PSS was defined as contrast staining outside the stent contour extending to 20% of the stent diameter, measured by quantitative coronary angiography. Morphologic classifications of PSS included mono-focal, multi-focal, segmental irregular-contour, and segmental smooth-contour as previously described. [16] We evaluated late acquired PSS in the current analysis, which was defined as that observed at follow-up or at the time of ST, but not at post-stenting.

### Intravascular Ultrasound Imaging and Analysis

IVUS images were acquired using commercially available imaging systems (ViewIT, Terumo; Galaxy and iLab, Boston Scientific) at the time of ST. According to the American College of Cardiology clinical expert consensus document on IVUS, 2 experienced physicians (Yamaji K and Kuramitsu S) carefully performed the qualitative assessment of IVUS images for ISA, presence of plaque rupture either inside or outside the stent, and stent fracture in a blinded manner regarding the timing of ST and hospitals. [27] ISA was defined as one or more stent struts separated from the vessel wall either with evidence of blood speckles behind the strut in a vessel segment not associated with any side branches or with occlusive thrombus between stent struts and the vessel wall. [28] In addition to the angiographic definition of stent fracture, stent fracture was also defined as complete or partial separation of stent segments observed by IVUS. [25], [26]

### Statistical Analysis

Categorical variables are expressed as number and percentages. Frequency analysis was performed with the chi-square test except for ordinal data. Ordinal data with more than 2 groups, such as Thrombolysis in Myocardial Infarction (TIMI) flow grade, was analyzed with Kruskal-Wallis test. Continuous variables are expressed as mean values ± SD or median values with IQR, and the differences were assessed by the unpaired t test. When comparing more than 2 groups, variables were assessed by using one way analysis of variance. All statistical analyses were performed with JMP 9.03 (SAS Institute, Cary, NC). All reported probability values were 2-sided, and probability values <0.05 were regarded as statistically significant.

## Results

### Patient and Lesion Characteristics

Mean age of the study patients at the time of ST was 67±10 years, and 40 patients (83%) were male. There was no significant difference in baseline patient and lesion characteristics among patients with EST, LST, and VLST, except for the type of DES (Table 1 and 2). All patients with VLST had first-generation DES thrombosis, while 8 patients (47%) of EST and 5 patients (71%) of LST had first-generation DES thrombosis. In patients with VLST, status of antiplatelet therapy at the time of thrombosis included DAPT in 12 patients (50%), aspirin only in 8 patients (33%), thienoprydine only in 3 patients (13%), and no antiplatelet therapy in 5 patients (21%), while 13 out of 17 EST patients (76%) and 3 out of 7 LST patients (43%) received DAPT at the time of thrombosis.

**Table 1:** Patient Characteristics at Time of Stent Thrombosis According to the Timing of Stent Thrombosis.

	EST (N = 17)	LST (N = 7)	VLST (N = 24)	P value
Days between the index procedure and ST	8.6±8.8	151±127	1445±548	
mean, interquartile range	5 (3–12.5)	61 (45–286)	1349 (1032–1886)	
Age, years	67±9	65±9	68±11	0.83
Male gender	16 (94)	5 (71)	19 (79)	0.30
Hypertension	13 (77)	6 (86)	16 (67)	0.56
Diabetes mellitus	11 (65)	5 (71)	12 (50)	0.48
Oral glucose lowering agents	5 (29)	0 (0)	5 (21)	0.27
Insulin	4 (24)	1 (14)	5 (21)	0.88
Current smoking	2 (12)	2 (29)	5 (21)	0.59
Lipid profile				
Total cholesterol, mg/dl	160±25.5	174±32.0	169±47.0	0.70
Triglyceride, mg/dl	94.8±46.4	131±104	98.4±51.6	0.39
HDL, mg/dl	50.8±14.0	47.4±11.3	41.9±12.6	0.10
LDL, mg/dl	91.3±21.3	98.0±21.7	101±33.4	0.59
Chronic renal failure	2 (12)	2 (29)	6 (25)	0.51
Hemodialysis	1 (5.9)	1 (14)	2 (8.3)	0.80
Left ventricular dysfunction (LVEF <40%)	3 (18)	1 (14)	2 (8.3)	0.67
Antiplatelet therapy				0.01
Dual antiplatelet therapy	13 (76)	3 (43)	12 (50)	
Aspirin only	3 (18)	0 (0)	8 (33)	
Thienopyridines only	0 (0)	0 (0)	3 (13)	
None	1 (5.9)	4 (57)	5 (21)	
ACE-I/ARB	10 (59)	4 (57)	10 (42)	0.51
Beta blocker	7 (41)	3 (43)	8 (33)	0.83
Statin	12 (71)	3 (43)	11 (46)	0.24
TVR before stent thrombosis	0 (0)	0 (0)	6 (25)	0.03

Values are expressed as means ± SD, median value with interquartile range, or number (%).

EST  =  early stent thrombosis, LST  =  late stent thrombosis, VLST  =  very late stent thrombosis, ST  =  stent thrombosis, HDL  =  high-density lipoprotein, LDL  =  low-density lipoprotein, LVEF  =  left ventricular ejection fraction, ACE-I  =  angiotensin-converting enzyme inhibitor, ARB  =  angiotensin receptor blocker, and TVR  =  target vessel revascularization.

**Table 2:** Lesion Characteristics at the Index Procedure and Time of Stent Thrombosis According to the Timing of Stent Thrombosis.

At the index procedure	EST (N = 17)	LST (N = 7)	VLST (N = 24)	P value
DES type				<0.001
First generation DES	8 (47)	5 (71)	24 (100)	
sirolimus-eluting stent	6 (35)	2 (28)	21 (87)	
paclitaxel-eluting stent	2 (12)	3 (43)	3 (13)	
Second generation DES	9 (53)	2 (29)	0 (0)	
everolimus-eluting stent	1 (5.9)	2 (29)	0 (0)	
zotarolimus-eluting stent	4 (24)	0 (0)	0 (0)	
biolimus-eluting stent	4 (24)	0 (0)	0 (0)	
Lesion location				0.39
Left anterior descending coronary artery	6 (35)	5 (71)	11 (46)	
Right coronary artery	5 (29)	1 (14)	9 (38)	
Left circumflex coronary artery	4 (24)	0 (0)	3 (13)	
Left main coronary artery	2 (12)	1 (14)	0 (0)	
Saphenous vein graft	0 (0)	0 (0)	1 (4)	
Bifurcation	8 (47)	2 (29)	9 (38)	0.67
Chronic total occlusion	3 (18)	1 (14)	2 (8.3)	0.67
Calcification	5 (29)	2 (29)	3 (13)	0.36
Ostium	7 (41)	1 (14)	6 (25)	0.34
Restenotic lesion	4 (24)	1 (14)	9 (38)	0.40
BMS restenosis	2 (12)	1 (14)	6 (25)	0.53
DES restenosis	0 (0)	0 (0)	2 (8)	0.35
Balloon angioplasty restenosis	2 (12)	0 (0)	1 (4)	0.47
Culprit lesion for ACS	6 (35)	3 (43)	7 (29)	0.90
ST-segment elevation myocardial infarction	4 (24)	2 (29)	3 (13)	
Non-ST-segment elevation myocardial infarction	1 (6)	0 (0)	1 (4)	
Unstable angina pectoris	1 (6)	1 (14)	3 (13)	
Multiple stents use	7 (41)	3 (43)	6 (25)	0.47
At stent thrombosis				
TIMI flow grade (pre)				0.31
3	2 (12)	1 (14)	0 (0)	
2	0 (0)	1 (14)	2 (8.3)	
1	0 (0)	0 (0)	2 (8.3)	
0	15 (88)	5 (71)	20 (83)	
TIMI flow grade (post)				0.35
3	17 (100)	7 (100)	22 (92)	
2	0 (0)	0 (0)	2 (8.3)	
1	0 (0)	0 (0)	0 (0)	
0	0 (0)	0 (0)	0 (0)	

Values are expressed as number (%).

EST  =  early stent thrombosis, LST  =  late stent thrombosis, VLST  =  very late stent thrombosis, DES  =  drug-eluting stents, BMS  =  bare-metal stents, ACS  =  acute coronary syndrome, and TIMI  =  thrombolysis in myocardial infarction.

### Angiographic and IVUS Findings

PSS was not observed in patients with EST and LST. In patients with VLST, PSS was found in 5 patients at the time of follow-up angiography (3 out of 19 patients) and/or at the time of ST (3 out of 24 patients) (Table 3). PSS morphology was mono-focal in 1 patient, multi-focal in 2 patients, and segmental irregular-contour in 2 patients. Of 24 patients with VLST, 15 patients (63%) underwent IVUS at the time of ST. We performed IVUS immediately after crossing the guidewire in 3 patients; after thrombus aspiration in 10 patients. We excluded two patients from the IVUS analysis: a patient who underwent IVUS after thrombus aspiration and balloon dilatation and that after additional stenting for the distal portion of the index stent. ISA was regarded as present in 9 patients with VLST (69%). Among them, clearly malapposed strut with evidence of blood speckles was observed in 5 patients (38%) and occlusive thrombus around the struts was present in 4 patients (31%). Finally, either PSS and/or ISA was identified in 12 (50%) out of 24 patients with VLST (Table 3). Stent fracture was identified in 5 (21%) patients with VLST (by both plain fluoroscopy and IVUS in 1 patient and only by IVUS in 4 patients). Among 12 patients with either PSS and/or ISA, 4 patients had stent fracture, while stent fracture was identified in 1 out of 12 patients without PSS or ISA. There was no evidence of plaque rupture in any patient who underwent IVUS. Of 24 patients with VLST, 8 patients underwent the index DES implantation for the restenotic lesions. There was no significant association for the presence of either PSS and/or ISA and the index DES implantation either for the de novo lesion or restenotic lesions (8 out of 16 patients [50%] with de novo lesions versus 4 out f 8 patients [50%] with restenotic lesions, P>0.99).

**Table 3:** Findings in VLST patients with either peri-stent contrast staining or incomplete stent apposition.

Age at ST(years)	Sex	Time to ST (days)	Indication for the index procedure	Antiplatelet therapy status at ST	Target lesion artery	DES type	Number of stents	ISR lesion	PSS	At ST	PSS morphology	IVUS	plaque rupture	Fracture	Eosinophils(per mm^2^, %)	Total WBC(per mm^2^)	Area (mm^2^)	Fibrous cap	Cholesterol crystal	Foamy macrophage
									Before ST			ISA								
82	Male	575	Stable AP	Aspirin	RCA	PES	1	N	-	N		Y	N	Y	175 (19.3)	906	0.725	N	N	N
52	Male	807	Stable AP	DAPT	RCA	SES	1	Y	N	N		Y	N	N	60 (7.6)	795	0.58	N	N	N
79	Male	809	Stable AP	None	RCA	PES	1	Y	N	N		Y	N	N	10 (1.1)	953	0.58	N	N	N
81	Male	827	STEMI	DAPT	SVG	SES	1	Y	Y	N	Multi-focal	-	-	N	86 (16.5)	517	0.725	N	N	N
63	Male	1033	Stable AP	Aspirin	LAD	SES	3	N	Y	Y	Segmental irregular	Y	N	N	307 (19.4)	1586	0.725	N	N	N
67	Female	1114	Stable AP	None	LAD	SES	1	N	Y	Y	Segmental irregular	-	-	Y	83 (13.9)	597	0.58	N	N	N
51	Male	1317	Stable AP	DAPT	RCA	SES	3	N	N	Y	Mono-focal	Y	N	Y	17 (1.9)	890	0.58	N	Y	Y
55	Female	1344	Stable AP	Aspirin	LAD	SES	1	N	N	Y	Multi-focal	-	-	N	47 (11.9)	390	0.58	N	N	N
68	Male	1929	Stable AP	Aspirin	RCA	SES	2	N	N	N		Y	N	N	135 (10.8)	1243	0.58	N	N	N
74	Female	2132	Stable AP	Clopidogrel	RCA	SES	1	Y	-	N		Y	N	Y	40 (11.7)	341	0.725	N	N	N
70	Male	2258	Stable AP	DAPT	LAD	SES	3	N	N	N		Y	N	N	302 (8.8)	3443	0.58	Y	Y	Y
71	Male	2691	Stable AP	Clopidogrel	LCX	SES	2	N	-	N		Y	N	N	171 (4.8)	3538	0.58	N	N	N

VLST  =  very late stent thrombosis, ST stent thrombosis, DES  =  drug-eluting stents, BMS  =  bare-metal stents, ISR  =  in-stent restenosis, PSS  =  peri-stent contrast staining, IVUS  =  intravascular ultrasound, ISA  =  incomplete stent apposition, WBC  =  white blood cell, A P  =  angina pectoris, STEMI  =  ST-segment elevation myocardial infarction, DAPT  =  dual antiplatelet therapy, RCA  =  right coronary artery, SVG  =  saphenous vein graft, LAD  =  left anterior descending artery, LCX  =  left circumflex artery, PES  =  paclitaxel-eluting stent, and SES  =  sirolimus-eluting stent.

### Histopathologic Findings of Aspirated Thrombi

**Figure 2:**
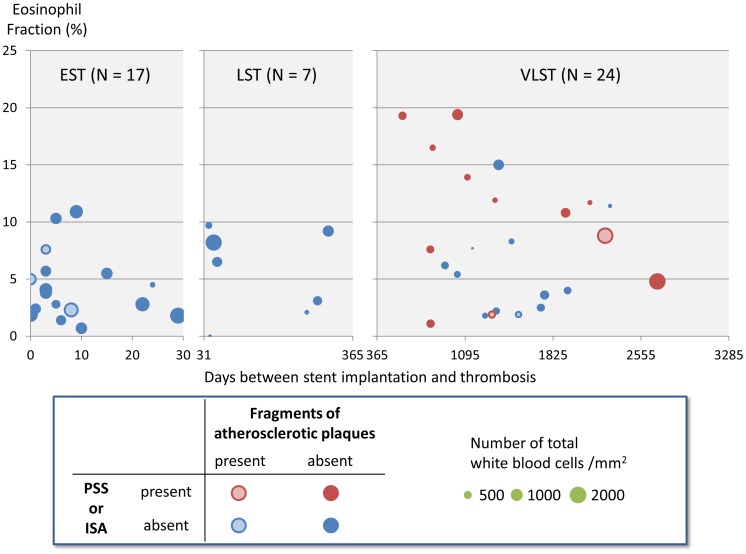
Bubble plots for eosinophil fraction in the aspirated thrombi and days between stent implantation and thrombosis in patients with and without PSS or ISA. Bubble size shows total white blood cell counts per mm^2^ in the aspirated thrombi. EST  =  early stent thrombosis, LST  =  late stent thrombosis, VLST  =  very late stent thrombosis, PSS  =  peri-stent contrast staining, and ISA  =  incomplete stent apposition.

**Figure 3:**
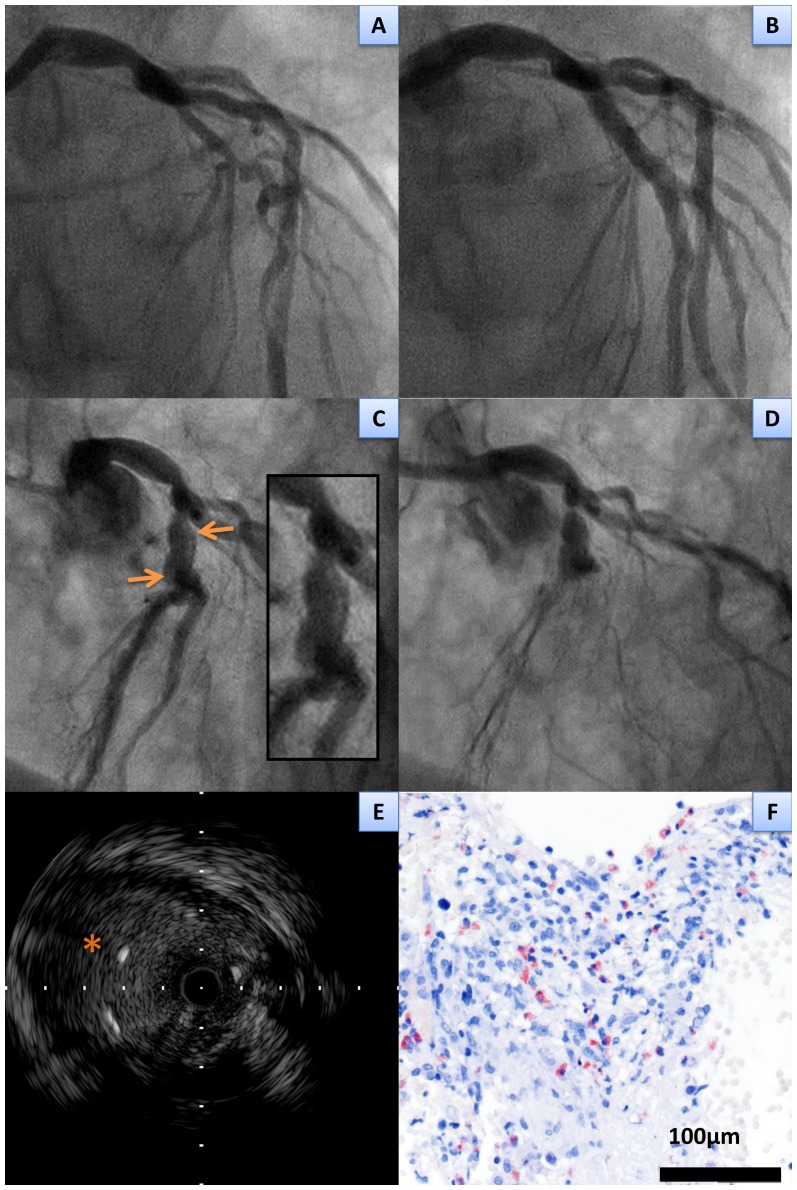
A representative case of very late stent thrombosis with eosinophilic infiltrates in the aspirated thrombus retrieved from lesions with peri-stent contrast staining and incomplete stent apposition. **A**, **B** Pre and post coronary angiography at the index sirolimus-eluting stents implantation (modified T stenting) demonstrated bifurcation lesion at the left anterior descending coronary artery. **C**, peri-stent contrast staining (**arrows**) was found by follow up angiography 813 days after the index procedure. **D**, very late stent thrombosis occurred at 1033 days after the index procedure. **E**, Intravascular ultrasound demonstrated intra-stent thrombus and incomplete stent apposition with positive arterial remodeling (**asterisk**). **F**, The histopathological image of the aspirated thrombus showed inflammatory infiltrate with eosinophils (19.4% out of total white blood cells) (Luna stain).

**Figure 4:**
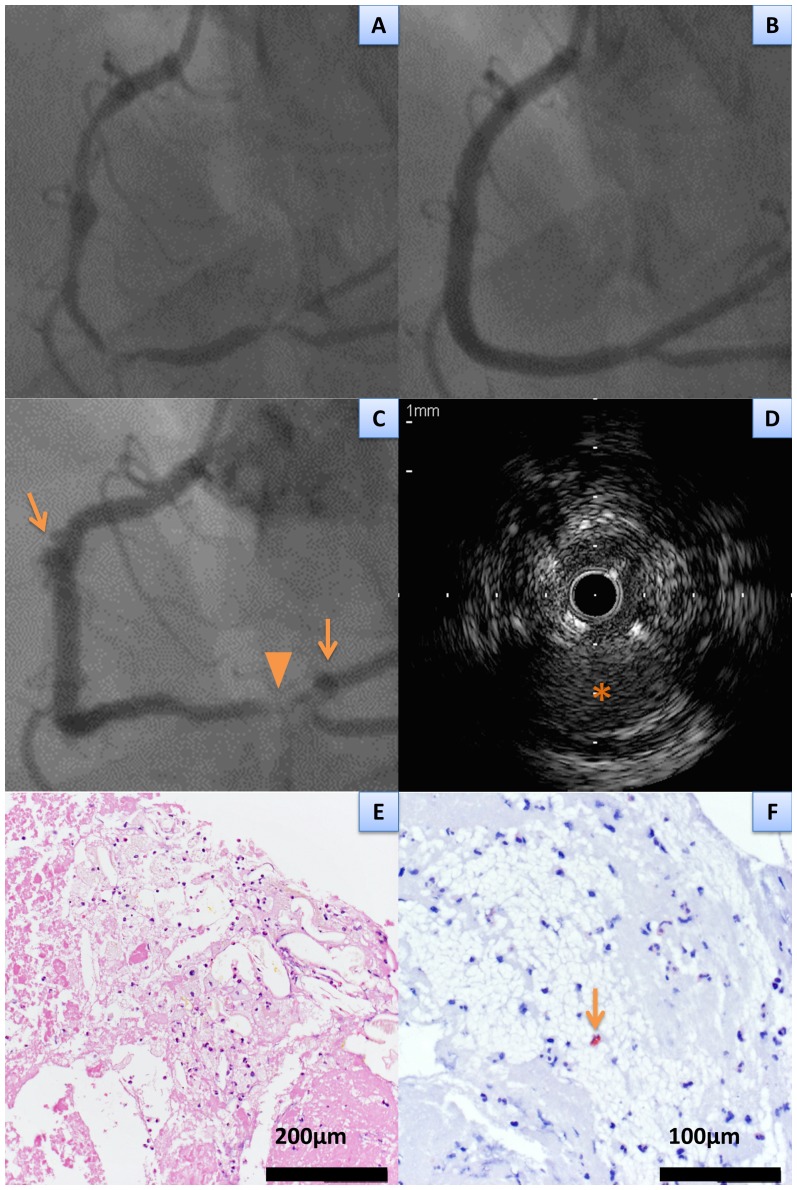
A representative case of very late stent thrombosis with fragments of atherosclerotic plaques retrieved from lesions with peri-stent contrast staining and incomplete stent apposition. **A**, **B** pre and post coronary angiography for 3 sirolimus-eluting stents implantation in the right coronary artery. **C**, very late stent thrombosis occurred at 1317 days after the index procedure. Peri-stent contrast staining (**arrows**) was found in the proximal and distal bifurcation lesions in the right coronary artery. Radiolucency (**arrow head**) suggesting the presence of thrombus was found in the distal bifurcation lesion. **D**, Intravascular ultrasound demonstrated intra-stent thrombus and incomplete stent apposition with positive arterial remodeling (asterisk). **E**, cholesterol crystals with foamy macrophages were observed in the aspirated thrombus (hematoxylin and eosin stain). **F**, eosinophils (arrow) were sparsely observed in the aspirated thrombus (1.9% out of total white blood cells) (Luna stain).

Eosinophil fraction in the aspirated thrombi was significantly higher in patients with VLST (8.2±5.7%) as compared with those with EST (4.3±3.0%) and LST (5.5±3.8%) (P = 0.03, Table 4 and Figure 2). In a total of 12 VLST patients with PSS and/or ISA, eosinophil fraction in the aspirated thrombi were significantly higher than those in 12 VLST patients without PSS or ISA (10.6±6.1% versus 5.8±4.1%, P = 0.03). A representative case demonstrating prominent eosinophilic infiltration and PSS was shown in Figure 3. Evidence for fragments of atherosclerotic plaques, including thin fibrous cap, foamy macrophages, or cholesterol crystals, was identified in 7 patients (15%). There was no significant difference in the prevalence of fragments of atherosclerotic plaques among patients with EST (N = 3 [18%]), LST (N = 1 [14%]), and VLST (N = 3 [13%]) (P = 0.9) (Table 4). Three VLST patients with an evidence for fragments of atherosclerotic plaque had relatively low eosinophil fraction (8.8%, 1.9%, and 1.9%) in the aspirated thrombus (Figure 4). Among the 5 patients with stent fracture, 3 patients had high eosinophil fraction (15.0±3.9%) associated with PSS and/or ISA, 1 patient had low eosinophil fraction (1.9%) with ISA, and 1 patient had low eosinophil fraction (2.5%) without PSS or ISA. There was no significant difference in the eosinophil fraction in the aspirated thrombi between patients who underwent index DES implantation for the de novo lesions and those for the restenotic lesions. (7.7±5.9% versus 9.4±5.3%, P = 0.49).

**Table 4:** Pathologic Findings in the Aspirated Thrombi According to the Timing of Stent Thrombosis.

	EST (N = 17)	LST (N = 7)	VLST (N = 24)	P value
Fragments of atherosclerotic plaques	3 (18)	1 (14)	3 (13)	0.90
Foamy macrophage	3 (18)	1 (14)	3 (13)	0.90
Cholesterol crystal	2 (12)	1 (14)	3 (13)	0.99
Thin fibrous cap	1 (6)	0 (0)	1 (4)	0.83
Eosinophil fraction	4.3±3.0%	5.5±3.8%	8.2±5.7%	0.03
Eosinophils per mm^2^	29.6±22.7	49.5±55.7	81.1±90.8	0.07
Total WBCs per mm^2^	714±276	681±619	901±854	0.37

Values are expressed as number (%).

EST  =  early stent thrombosis, LST  =  late stent thrombosis, VLST  =  very late stent thrombosis, ST  =  stent thrombosis, and WBC  =  white blood cell.

## Discussion

The main findings of the present study were as follows; (1) Eosinophil fraction in the aspirated thrombi was significantly higher in patients with DES VLST as compared with those with EST and LST; (2) Eosinophil fraction in the aspirated thrombi in patients with DES VLST was significantly higher in patients with PSS and/or ISA as compared with those without PSS or ISA; and (3) Evidences for fragments of atherosclerotic plaques were relatively uncommon in patients with DES VLST.

IVUS studies after SES implantation revealed a higher incidence of ISA with DES compared with BMS (8.7% versus 0.0%, P<0.05). [28] From the several IVUS studies, the prevalence of ISA at the time of VLST was high (52%–74%). [10], [12]–[14] In the present study, we found PSS and/or ISA in 12 (50%) out of 24 lesions of VLST. The prevalence of PSS and ISA were consistent with the previous reports. The RESTART (Registry of Stent Thrombosis for review And Re-evaluaTion) was a Japanese large registry of SES-associated ST, [29] and the angiographic substudy demonstrated that PSS was present in 34.1% of patients with SES-associated VLST. [30] Recently, Imai et al. reported that PSS was found within 12 months after SES implantation in 194 lesions (2.5%) out of 7838 lesions, and cumulative incidence of VLST at 4 years was significantly higher in lesions with PSS (5.3%) as compared with those without PSS (0.7%, P<0.001). [17] Therefore, ISA and PSS might represent abnormal vessel wall responses to DES, which predisposed to VLST. However, the pathophysiologic mechanisms of ISA and PSS formation leading to VLST have not been yet adequately clarified.

Cook et al. reported that the number of eosinophils in the aspirated thrombi at the time of DES VLST correlated with the extent of stent malapposition in relatively small number of patients (10 patients [11 lesions]). [14] In the current study evaluating larger number of patients with DES VLST, eosinophil fraction in the aspirated thrombi was significantly higher in patients with VLST as compared with those with EST and LST. Furthermore, eosinophil fraction in the aspirated thrombi in patients with DES VLST was significantly higher in patients with PSS and/or ISA as compared with those without PSS or ISA. Higher eosinophil fraction in the aspirated thrombi might be attributable to localized hypersensitivity vasculitis. Indeed, previous human pathologic studies suggested that hypersensitivity vasculitis in response to SES caused intimal damage leading to vascular surface defect with PSS and/or ISA, [9], [15] and long-lasting severe hypersensitivity vasculitis might lead to further medial disruption and destruction of vessel wall with aneurysmal dilatation. [31] Localized hypersensitivity vasculitis was a major underlying mechanisms of VLST in patients with positive remodeling and mal-apposition of the stent struts. In contrast, there was a postmortem case report demonstrating late ISA by focal positive vessel remodeling caused by medial necrosis without hypersensitivity reaction. [32] Medial necrosis with medial smooth muscle cell depletion could also be a possible mechanism for positive remodeling and mal-apposition of the stent struts.

The mechanisms of DES VLST could be multi-factorial. In a human post-mortem pathologic study, neoatherosclerosis was a frequent finding in the DES-treated lesions and occurred earlier than in the BMS-treated lesions. [22] Therefore, disruption of neoatherosclerosis inside the stents was suggested to be one of the important underlying mechanisms of DES VLST. In the previous single-center study, we evaluated the evidence for fragments of atherosclerotic plaques, such as foamy macrophages, cholesterol crystals, and thin fibrous cap, in the aspirated thrombi in patients with BMS thrombosis. [23] Fragment of atherosclerosis was observed in 13 out of 42 patients [31%] with BMS VLST in the previous study, while it was observed only in 3 out of 24 patients [13%] with DES VLST in the current study. Although the time intervals between the index procedure and VLST was significantly shorter in patients with DES VLST as compared with those with BMS VLST (5.5±3.0 years versus 4.0±1.5 years), it was surprising that the prevalence of fragment of atherosclerosis was lower in patients with DES VLST as compared with BMS VLST, given the high prevalence of in-stent neoatherosclerosis reported in the DES-treated lesions. [22] Further human pathologic and/or imaging studies are important to investigate the possible role of in-stent neoatherosclerosis as one of the mechanisms of DES VLST.

Although it has not been fully clarified whether DAPT beyond 1 year could decrease the incidences of VLST, [33] 12 out of 24 patients (50%) underwent DAPT at the time of VLST in this study. From the long-term follow-up of j-Cypher registry, which was a nationwide Japanese registry of patients with SES implantation, 43.9% of patients underwent DAPT at 5 years. [4] Physicians might prolong the duration of DAPT after the observation of abnormal findings at the scheduled follow-up angiography, especially in patients with the first generation DES.

### Study Limitations

The present study has several limitations. (1) The number of patients was small to fully elucidate the prevalence of inflammatory cell infiltrates and evidence of atherosclerosis in the aspirated thrombi in patients with DES VLST. (2) It was not certain whether the eosinophils in the aspirated thrombi were actually derived from the stented vessel wall. As in the previous study, [23] absence of fragments of atherosclerotic plaque does not necessarily mean absence of neoatherosclerosis inside the stents because thrombus aspiration could not fully retrieve the components of the arterial wall. (3) All cases of VLST in the current study occurred only with first generation DES. This study could not provide information on the newer generation DES thrombosis. First-generation DES is no longer used in the clinical arena. However, there are millions of patients who had already received first-generation DES. (4) We did not perform IVUS for all patients, and no patient underwent optical coherence tomography. IVUS does not have enough resolution to provide data on neoatheroscleosis, plaque rupture or thickness of a thin-cap fibroatheroma. Furthermore, even though it has enough resolution to measure vessel, stent and lumen area, we could not fully assess those measurements due to mural thrombi. We could not fully discriminate progressive in-stent restenosis with superimposed thrombus from stent thrombosis. (5) The proportion of patients who underwent index DES deployment for the restenotic lesions was relatively high in the current analysis. The risk of thrombosis of two stent layers could be higher than one stent layer. Even though there was no significant difference in the eosinophil fraction in the aspirated thrombi between patients who underwent index DES implantation for the de novo lesions and those for the restenotic lesions, overlapping stent could be associated with late changes around the stent struts. However, the number of patients in this analysis was small for the subgroup analysis to elucidate the association of restenotic lesions and underlying mechanisms of stent thromobosis.

## Conclusions

Eosinophil fraction in the aspirated thrombi was significantly higher in patients with DES VLST as compared with those with EST and LST. Evidences for fragments of atherosclerotic plaques were relatively uncommon in patients with DES VLST.
